# On the growth mode of two-lobed curvilinear graphene domains at atmospheric pressure

**DOI:** 10.1038/srep02571

**Published:** 2013-09-03

**Authors:** Kitu Kumar, Eui-Hyeok Yang

**Affiliations:** 1 Department of Mechanical Engineering, Stevens Institute of Technology, Hoboken, New Jersey 07030

## Abstract

We demonstrate the chemical vapor deposition (CVD) growth of 2-lobed symmetrical curvilinear graphene domains specifically on Cu{100} surface orientations at atmospheric pressure. We utilize electron backscattered diffraction, scanning electron microscopy and Raman spectroscopy to determine an as-yet unexplored growth mode producing such a shape and demonstrate how its growth and morphology are dependent on the underlying Cu crystal structure especially in the high CH_4_:H_2_ regime. We show that both monolayer and bilayer curvilinear domains are grown on Cu{100} surfaces; furthermore, we show that characteristic atmospheric pressure CVD hexagonal domains are grown on all other Cu facets with an isotropic growth rate which is more rapid than that on Cu{100}. These findings indicate that the Cu-graphene complex is predominant mechanistically at atmospheric pressure, which is an important step towards tailoring graphene properties via substrate engineering.

The unique electronic and mechanical properties of graphene have made this 2-D material a test-bed for novel laboratory physics experiments as well as a contender for applications such as transparent electrodes^1^, supercapacitors^2^, high-speed electronics^3^, plasmonics^4^,^5^, photovoltaics^6^,^7^, photodetectors^8^,^9^, and large scale transistors^10^. The majority of physics research has been conducted on mechanically exfoliated graphene, but has limitations in practical applications due to the small areas achieved by this fabrication method. Therefore, efforts have focused on large-area growth techniques such as epitaxy on various substrates^11^,^12^ and metal-catalyzed thermal decomposition of hydrocarbons. Growth using this latter method, also known as CVD especially on copper (Cu)^13^, has generated much interest due to the low cost of the Cu foil, ease of large-area growth at relatively low temperatures and ease of transfer to various substrates for device applications^14^,^15^. Studies of the fundamental CVD processes driving graphene growth such as system thermodynamics and growth kinetics including surface diffusion^16^, active species adsorption/desorption^17^ and edge-oriented crystal growth^18^, have been conducted to benefit graphene synthesis by reducing grain boundaries and defects such that CVD graphene quality can approach that of mechanically exfoliated graphene. Thus, the dynamics of the graphene/Cu interface during growth are the subject of intensive examination. Of great interest are the shapes and alignment of graphene domains^16^,^19^,^20^,^21^,^22^ on Cu surfaces which directly influence the mechanical, chemical, and electrical properties of the coalesced polycrystalline graphene film^23^.

A recent work showed that monolayer graphene grown on single crystal Cu(111) under ultra high pressure chemical vapor deposition formed a continuous sheet with few grain boundaries, while that grown on single crystal Cu(100) was non-continuous and displayed exposed domain edges poorly aligned with the underlying Cu direction^24^. The monolayer graphene grown under more typical low pressure (LPCVD) conditions usually has a 4-lobed, 4-fold-symmetric domain^21^,^25^, though large-area, dendritic growths have been demonstrated^13^. The 4-lobed domains growth mode was shown to be dominated by edge kinetics with a growth velocity dependent on the orientation of the graphene with respect to the underlying single crystal Cu(100) substrate^21^. In addition, studies in atmospheric-pressure chemical vapor deposition (APCVD) graphene synthesis have demonstrated that the underlying crystal orientation in the typical polycrystalline Cu plays a greater role in domain growth kinetics and thus domain shape, size, and nucleation density than originally thought^11^,^26^. Typical domain shapes from literature in APCVD synthesized graphene are 6-fold symmetric hexagons for both monolayer^27^,^28^,^29^ and multilayer graphene^27^,^30^. However, regardless of Cu orientation, the growth mode of orientation-dependent domains remains largely unexplored.

Here we show the APCVD growth of 2-lobed curvilinear monolayer and bilayer domains exclusively with 1-fold symmetry on Cu{100} surfaces. This finding reveals hitherto unexamined growth kinetics, which are modulated to a greater extent than previously reported by surface energy anisotropy of Cu at atmospheric pressure. To elucidate the growth mode of this domain shape with respect to the Cu surface, we use a combination of electron backscattered diffraction (EBSD), scanning electron microscopy (SEM) and Raman spectroscopy. We discuss the effects of diffusion limited growth which affects the symmetry of these domains and also examine the angular dependence of the graphene axis due to surface anisotropy which controls the curvilinear morphology to fully determine the effect of the Cu lattice on growth kinetics. We show that this anisotropic growth mode is not pronounced on higher index Cu surfaces, since hexagonal domains are found under the same APCVD conditions on surfaces which are not {100}. Furthermore, we experimentally show that the anisotropic growth rate of the curvilinear domain is slower than the isotropic growth rate of hexagonal/polygonal domains and attribute this to strain effects from lattice mismatch. Finally, we discuss how Cu surface energy may influence the bilayer growth mode, inducing similar growth kinetics, by straining of the intermediary monolayer graphene sheet.

## Results

**Figure 1:**
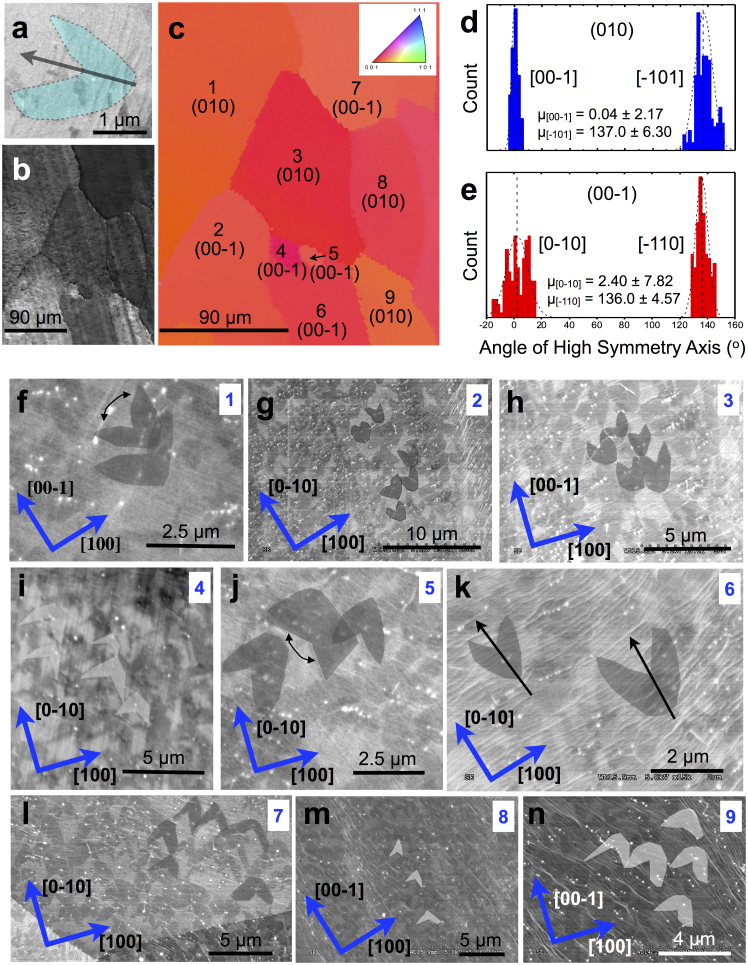
SEM and EBSD images of Cu grain effect on graphene domain shape. (a) Representative SEM image of a 2-lobed curvilinear monolayer domain grown under APCVD. (b) Low resolution SEM image of a high purity copper area with 2-lobed curvilinear domain growth. (c) Inverse pole EBSD map of the area in (b) indicating that the 2-lobed domains grow predominantly on the (010) and (00–1) surfaces. Inset to (c) is the color key used to determine surface orientation. (d) Alignment of the high-symmetry axis of domains on Cu(010) is along primarily along [00–1] and [−101] whereas in (e) the high-symmetry alignment on Cu(00–1) is primarily along [−110] with a secondary alignment along [0–10]. The dashed line is a normal fit to the data, where n = 100 and the values presented as error are the standard deviations from the mean, μ. (f-n) SEM images of false-color highlighted monolayer 2-lobed graphene domains corresponding to the growth on each Cu grain mapped in (c).

We investigated graphene growth under conditions chosen to elucidate the mechanism of anisotropic growth. Flow rates of 10 sccm methane (CH_4_) and 30–70 sccm H_2_ in 1000 sccm Ar were maintained during the growth phase, which was terminated after 12 s or 25 s to examine individual domains before they coalesced into a continuous film. Figure 1a provides an SEM image of an isolated 2-lobed curvilinear structure after 25 s of growth. These distinctive morphologies were grown only on the Cu{100} surfaces (Figure 1c) of high purity Cu (99.999% Alfa Aesar). The high symmetry axis of the domains was found to be well aligned, as shown in Figure 1d,e, along [00–1] and [−110] with secondary alignment along [−101] and [0–10], respectively. The high-symmetry axis was also found to be well aligned to the perpendicular intersect of Cu surface steps (Figure 1k,n). On Cu{100}, domain growth was found to remain unbroken across rolling striations, surface steps, and grain boundaries, though these surface features increased graphene nucleation density and played a role in morphology variation. Additionally, vicinity to other graphene domains had well-defined effects on single lobed morphology. The growth and presence of the curvilinear domain shape under APCVD conditions is puzzling; we elucidate the origin of this shape and growth mode below.

## Discussion

LPCVD studies often produce lobed or flower-like graphene domains^31^ on Cu(100) orientations. In these cases, growth has been shown to be limited by angularly dependent edge kinetics, *i.e*., alignment of the fast growth direction of individual lobes with both the slow growth direction and Cu orientation produces 4-lobed morphologies, such that the flakes formed are not single crystals^21^. On the other hand, in APCVD growth, the hexagon/polygonal single crystal domain morphologies typically reported are limited by edge selectivity during growth, wherein the carbon attachment to only armchair edges is energetically stable, thus converting these edges to the zigzag orientation^32^. The six-fold symmetric growths reported in these studies is largely independent of Cu surface orientation, except in one case where the growth temperature was reduced, thereby growing 2-fold symmetric rectangular domains on only Cu(111)^33^. However, in that study, the growth mechanism was not clearly elucidated.

**Figure 2:**
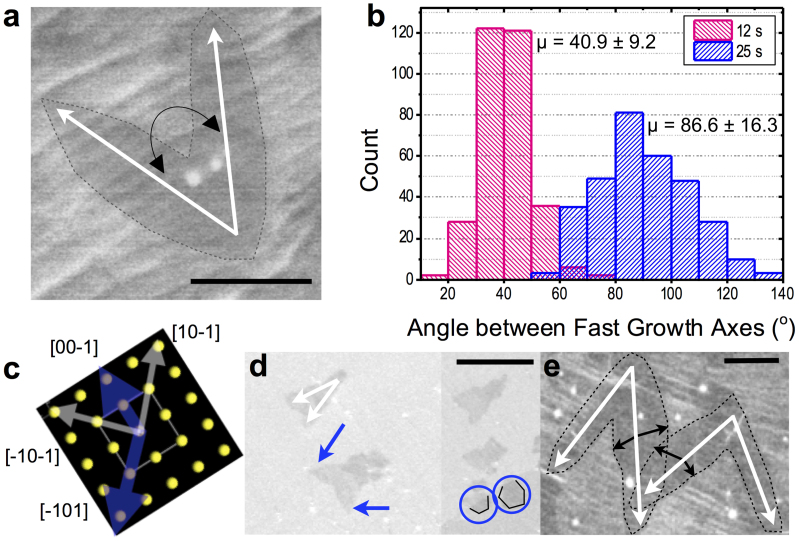
SEM images and data on 2-lobed curvilinear monolayer domain evolution. (a) A representative 25 s growth, 2-lobed curvilinear domain on Cu(010), with fast growth axes as indicated by white arrows. The curved black arrow represents the relative angle between the fast growth axes (lobes). (b) Histogram of angle between the fast growth axes of the curvilinear domains on Cu{100} surfaces after 12 s and 25 s of growth with mean values of 40.9° and 86.6°, respectively. n = 317 and the values presented as error are the standard deviations from the mean, μ. (c) Image of the (010) surface as in (a) with blue arrows denoting Cu directions, [00–1] and [−101], upon which the domains' high symmetry axes typically align. Grey arrows indicate direction of lobe growth, typically along [−10–1] and [10–1], if the high symmetry axis is aligned to [00–1]. Evolution of domain morphology between (d) 12 s and (e) 25 s of growth. Fast growth axes marked with white arrows. (d) Appearance of branching in the precursor to the two lobed structure is indicated with blue arrows. Morphology change from curvilinear to hexagonal over a grain boundary is marked with blue circles. (e) Increase of slow growth rate as the fast growth front of one domain approaches another. Based on percentage of Cu covered by the graphene domain between 12 s and 25 s of growth, a growth velocity of 1.13 ± 0.67 μm/s is calculated in the purely fast direction. Scale bars are (a) 1 μm, (d) 0.75 μm and (e) 2.5 μm.

In our study, the distinct case of curvilinear growth specifically on the Cu{100} facets may be influenced by two causes. One is the ratio of CH_4_:H_2_ pressure which, in the case of the experiments presented here, is higher than typically found in literature. According to Vlassiouk *et al.*^27^, at a fixed CH_4_ partial pressure, greater H_2_ partial pressures would result in a distinct hexagonal shape of domains with zigzag edges since hydrogen etching of graphene is most efficient along this orientation^27^,^34^. In these studies, carbon concentrations are in the parts per million, whereas our experiments have orders of magnitude greater CH_4_ concentrations as described above. Therefore, we are operating in a different growth regime, and thus hydrogen etching can be ruled out as the predominant mechanism for the curvilinear shape. The second cause is more complex and involves several growth kinetic processes, indicating an unstudied growth mode on Cu(100) at atmospheric pressure. To examine this growth mode, we scrutinize the following phenomena:

1. Mobile carbon atoms at the Cu surface can diffuse along one of the four equivalent lattice directions of the substrate which would extend the domain growth along one of these directions^35^. Indeed, graphene has already been proven to align in two directions on Cu(100) under both LPCVD and APCVD^22^,^26^,^35^,^36^ though the angle between the two directions can be smeared anywhere from 30–90° depending on the presence of step bunches or other surface interruptions. In our work, the two fast growth axes of the curvilinear domains (Figure 2a) are separated by a mean angle of 86.6° relative to each other (Figure 2b). Coupled with the alignment of the domain high symmetry axes with either [00–1] or [−101] on Cu(010) and either [−110] or [0–10] on Cu(00–1) indicates that the two fast growth fronts are most efficient along the <101> (Figure 2c) or <100> directions. This efficiency is attributed to surface energy anisotropy stemming from electron density correlations between the Cu lattice directions with highest atomic density and the graphene fast growth axes^26^.
2. If the domain growth is diffusion limited, *i.e.* directly influenced by surface energy anisotropy, the observed shape of a curvilinear lobe should be well fit to the function describing the angular dependence of the fast growth velocity to surface anisotropy^21^. Here, the velocity of the fast growth axis depends on the relative orientation of the graphene and Cu^21^. The model is described as 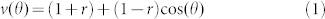
where *v* is the growth velocity, *θ* is the angle between the edge normal and slow growth direction, and *r* is the ratio of the velocities in the slow and the fast directions and is the anisotropy factor for the 4-fold symmetric Cu(100) surface. The purely fast axis mean velocity was determined to be 1.13 ± 0.67 (s.d.) μm/s from domain enlargement between 12 s and 25 s growths (Figure 2d,e respectively). Using the well-known anisotropy factor *r* = 0.25, which predicts a sharp tipped lobe and is prevalent in our domains as seen in Figure 2a, we found a close fit with our curvilinear shape (Supplementary Figure S1). This model demonstrates that the distinctive shape of the graphene lobes on Cu{100} emerges from a growth velocity which is indeed angularly dependent on surface anisotropy. Therefore, in the regime of high CH_4_:H_2_ growth, growth is dominated by surface diffusion and graphene orientation dependent kinetics, in sharp contrast to the reported APCVD 6-fold growth *via* edge selectivity^32^. We attribute the presence of the 1-fold symmetry with 2-lobes (rather than 4-lobes) to growth downhill the steps perpendicular to the [00–1] and [−110] directions Upward growth is prevented here due to the higher activation energy^37^ required for diffusion. We also note that as the fast growth front approaches adjacent domains (Figure 2e), the axis is not deflected; rather, the slow growth velocity increases in that direction. The cause of this effect is unclear, but accounts for the substantial variations from the mean lobe-to-lobe angle in 25 s growth compared to 12 s growth as in Figure 2b.

**Figure 3:**
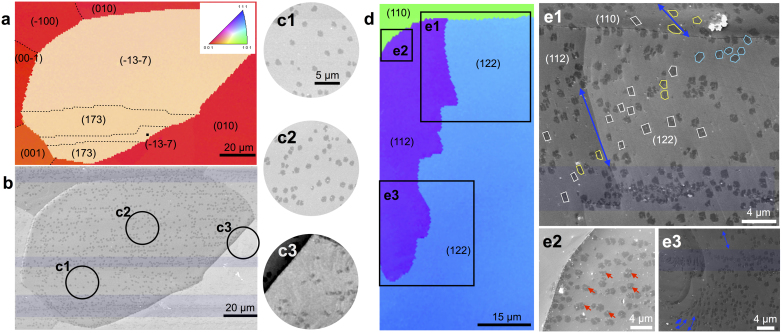
Graphene domain shape dependence on Cu orientation, 12 s growth. (a), (d) Inverse pole EBSD maps of predominantly non-(100) Cu facets. Inset to (a) is the color key used to determine surface orientation. (b), (c1-3), (e1-3) SEM images show that, under atmospheric pressure, hexagonal domains with predominantly isotropic growth trajectories are prevalent on high-index Cu facets such as (173), (122), and (112), with no evidence of the 2-lobed domains. (c1-3) SEM images of graphene domains whose low nucleation density is dependent on carbon concentration across the surface. (c3) Nucleation density of precursors to the 2-lobed curvilinear domain on (010) is lowest, confirming surface diffusion as the limiting factor in curvilinear growth. (e1-3) Details of high energy nucleation sites such as rolling striations (blue highlight) and nanoparticles (red arrows). (e1) Straining of growth trajectories due to surface steps (blue arrows) create morphological changes varying from predominantly isotropic growth domains (cyan hexagons) to moderately strained growth (yellow polygons) to 2-fold symmetric strained growth (white rectangles).

We now show that 6-fold symmetric graphene growth occurs on all other Cu surfaces measured. Figure 3 details 12 s growths on higher index Cu facets (Figure 3a,d). Here, the domains largely nucleate on rolling-induced striations (and this density remains the same across the Cu facets) or steps and nanoparticles^27^, all energetically favorable points which reduce the activation energy for adsorption of carbon species^38^. However, in locations where these surface interruptions do not exist, (Figure 3c,1–2), nucleation density can be assumed to be directly proportional to carbon concentration on the surface. The low nucleation density on the high index Cu surfaces and even lower density on Cu(010), further confirms that growth is surface diffusion limited^39^. On higher index Cu, the edges of the graphene domains appear to be randomly oriented. However, substantial morphology change occurs from hexagonal to rectangular shape (Figure 3e,1) as growth trajectories are strained^39^ parallel to the surface steps.

**Figure 4:**
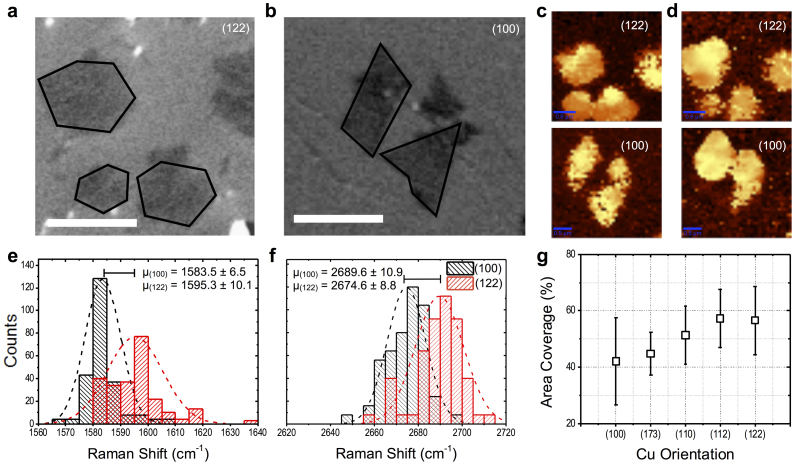
Strain and area coverage of graphene monolayer domains. (a), (b) SEM images of graphene domains on Cu(122) and (100), displaying isotropic polygonal and the precursor to the 2-lobed curvilinear graphene domains, respectively. (c), (d) Spatially resolved Raman spectroscopy maps of the G and G' band energies, respectively, from the specimens in (a) and (b). Qualitatively, the greater intensity of the signal from the domains on (122) indicate a greater band energy than in the domains on (100). (e), (f) Representative Raman energy histograms of the G and G' band energy of similar samples on these Cu facets, respectively. The decrease in G and G' energies indicate greater strain in the (100) samples when compared to the blueshift in the (122) facets. The dashed lines are normal fits to the data, where n = 300 and the values presented as error are the standard deviations from μ. (g) Area coverage of graphene domains on various Cu facets for 12 s of growth time. The higher indices have greater graphene coverage, thus confirming that isotropic growth trajectories are faster than non-isotropic trajectories as in the case of the 2-lobed domain. Scale bars in (a), (b) and (c), (d) are 1 μm and 0.5 μm, respectively.

As mentioned above, electron density correlation between the graphene and Cu direction is fundamental to growth along specific axes. That is, 2p C orbitals hybridize with the 3d Cu orbitals only in the directions which minimize lattice mismatch. Despite this growth efficiency, substantial strain is attributed to graphene grown on Cu(100) due to the sizeable lattice mismatch of a 6-fold symmetric crystal growing on a 4-fold symmetric substrate^40^ and can be visualized through Moire patterns^24^,^41^. The presence of strain can be determined using Raman spectroscopy^28^,^40^. In Figure 4c,d, the intensity of the Raman map of the G and G' full width-at-half-maximum (FWHM) is larger on the (100) surface than on (122) surface. Secondly, the Raman energy histograms (Figure 4e,f), of 12 s growth domains show substantial non-uniform strain on Cu(100) due to decrease of the Raman energy. Comparing this to the blue-shifts in the Raman bands of the hexagonal domains grown on Cu(122) clearly confirms the sizeable lattice mismatch of graphene on Cu(100), further supporting the case for the surface anisotropy modulated growth of 2-lobed curvilinear domains. Because the lattice mismatch minimization is more efficient on the higher energy Cu indices, it follows that graphene growth on these facets is more isotropic and faster than on the Cu(100). Indeed, our experiments corroborate this in Figure 4g; graphene coverage of the underlying Cu facet is shown to increase as the Cu index shifts away from the {100} orientation.

**Figure 5:**
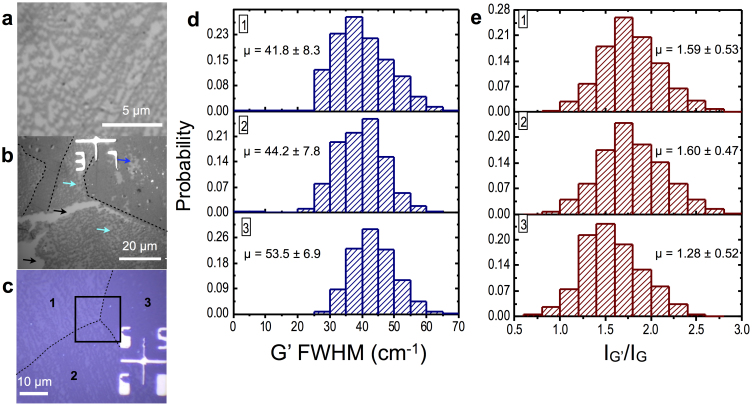
Evidence of Cu orientation influence on bilayer graphene growth. (a) Optical image of dense bilayer 2-lobed curvilinear graphene domains atop monolayer graphene transferred to 90 nm SiO_2_ substrate. (b) Optical confirmation of multilayer growth. Black arrows indicate tear in graphene sheet and underlying SiO_2_. Cyan arrows indicate 2-lobed multilayer domains. Cu facet influence is seen on bilayer surface nucleation and diffusion kinetics, through the intermediary monolayer, as the areas marked by the dotted lines are regions of fast, isotropic growth. The blue arrow indicates a single hexagonal graphene domain. (c) Optical color image of transferred multilayer-atop-monolayer graphene with a triple point region of varying Cu facets, (100) in regions 1 and 2 and a high-index facet in region 3. Scale bar is 10 μm. (d,e) Histograms of G' FWHM and I_G'_/I_G_ data extracted from the area marked by the black square in (c) indicating bilayer growth modes similar to monolayer graphene. Regions 1 and 2, which have lower bilayer domain nucleation density and coverage than the bilayer in region 3 (high index) also have lower G' FWHM and higher I_G'_/I_G_ indicating less presence of bilayer material. As in the case of monolayer growth, the slower bilayer curvilinear domain anisotropic growth can be attributed to strain effects. n = 500 and the values presented as error are the standard deviations from the mean, μ.

Straining of the monolayer graphene lattice may lead to interesting kinetics when it comes to the growth of bilayers. Currently, the nature of the growth mechanism of bilayer graphene on monolayer under APCVD is not extensively studied^42^,^43^. Here, by extending the growth time from 25 s to 1 min under the high CH_4_:H_2_ regime, we observe the coalescence of the monolayer graphene domains to a continuous sheet and the growth of secondary domains atop this sheet. Interestingly, the 2-lobed curvilinear shape is present in these bilayer domains (Figure 5a,b) indicating similar kinetics of formation. The bilayer domains were not clearly visible under SEM, therefore the graphene sample was etch-released from the Cu substrate and transferred to 90 nm SiO_2_ wafers for further analysis. We hypothesize that the growth process which occurs during monolayer growth *i.e.* surface diffusion and the graphene orientation-Cu direction dependent reactions are the same or similar during secondary layer growth. This strongly indicates that the Cu potential energy surface isotropy or anisotropy is transmitted to the intermediary monolayer which influences approaching carbon species^30^. Though the effect of strain cannot be determined adequately since Raman spectra for polycrystalline graphene varies from single crystal graphene^44^ and since the transfer process dopes the graphene^45^ which further affects the Raman bands, we note that Cu facet influence affects the nucleation density and growth rates of bilayer graphene in similar fashion to monolayer as evidenced in Figure 5b,c. The 2-lobed bilayer domains grown on Cu{100} have lower nucleation density and smaller sizes than their hexagonal counterparts indicating slow, strained growth. Indeed, data extracted from the G' FWHM, G' and G intensity Raman maps (Supplementary Figure S2) of the region indicated in Figure 5c confirms greater bilayer coverage (Figure 5d,e) in areas of fast isotropic growth by the greater incidence of broadened G' band FWHM and lower I_G'_/I_G_ intensity ratio. This result elucidates a similar strained growth mode of as-grown large area bilayer graphene on Cu{100} facets with high surface energy anisotropy, leading to interesting physics such as energy dispersion^46^ and bandgap tuning^47^ with applications in photonics devices such as photodetectors^8^,^9^ and photovoltaic cells^6^,^7^.

In summary, we have presented an analysis of a novel 2-dimensional graphene domain growth on Cu{100} facets whose unusual 2-lobed morphology and growth mode are controlled by the underlying surface orientation. We have shown that under the high CH_4_:H_2_ regime, the 2-lobed shape is largely controlled by surface diffusion and graphene orientation dependent kinetics, which are both influenced by the Cu(100) surface energy modulation along the high atomic density directions <101> and <100>. We have further shown that this growth mode rate is lower than the typical isotropic growth on higher index Cu facets, indicating the need for precise control of Cu orientation to grow high quality monolayer graphene. Finally, we have found that the growth of bilayer domains is similar to the monolayer growth mode and attribute this similarity to lattice straining of the intermediary graphene sheet. These findings indicate a hitherto unexplored anisotropic growth mode of graphene at atmospheric CVD conditions. Therefore, this work represents an important step in the fundamental understanding of the APCVD growth mechanisms for graphene as influenced by the underlying Cu orientation, which in turn will advance graphene synthesis for large-area applications.

## Methods

The CVD graphene samples employed in this work were grown at atmospheric pressure on 25 μm thick Cu foils (Alfa Aesar, 99.999% purity) in a quartz tube furnace at 1000oC with 1000sccm of Argon, 30–70 sccm of H_2_ and 10 sccm of CH_4_ flowing during growth. To transfer graphene, 95,000 molecular weight PMMA (Sigma-Aldrich) dissolved to 4% in anisole was spin-coated on the graphene-Cu stack at a speed of 4000 rpm for 1 min followed by 1000 rpm for 1 min (acceleration of 1000 rpm/s). The resulting PMMA thickness was approximately 50 nm. The sample was then dried at 25°C in laboratory ambient air for 12 hrs after which it was placed in a citric acid etchant bath (Transene, Inc) to remove the underlying Cu. The PMMA-graphene stack placed in two successive water baths of 12 hrs each and then transferred to a 90 nm SiO_2_/Si substrate.

## Author Contributions

K.K. designed and carried out the experiments, performed data analysis and wrote the manuscript. K.K. and E.H.Y. discussed the results, reviewed the manuscript, and have given approval to the final version of the manuscript.

## Supplementary Material

Supplementary Information Supplementary Information
