# Supplementary material for: On the growth mode of two-lobed curvilinear graphene domains at atmospheric pressure
Source: Sci Rep. 2013 Sep 3;3:2571. doi: 10.1038/srep02571 (PMC3759841; doi:10.1038/srep02571)
Supplement: Supplementary Information [file srep02571-s1.pdf]

Supplementary Information for

On the growth mode of two-lobed curvilinear graphene domains at atmospheric pressure

Kitu Kumar and Eui-Hyeok Yang*

Department of Mechanical Engineering, Stevens Institute of Technology, Hoboken, NJ 07030, USA

*email: eyang@stevens.edu

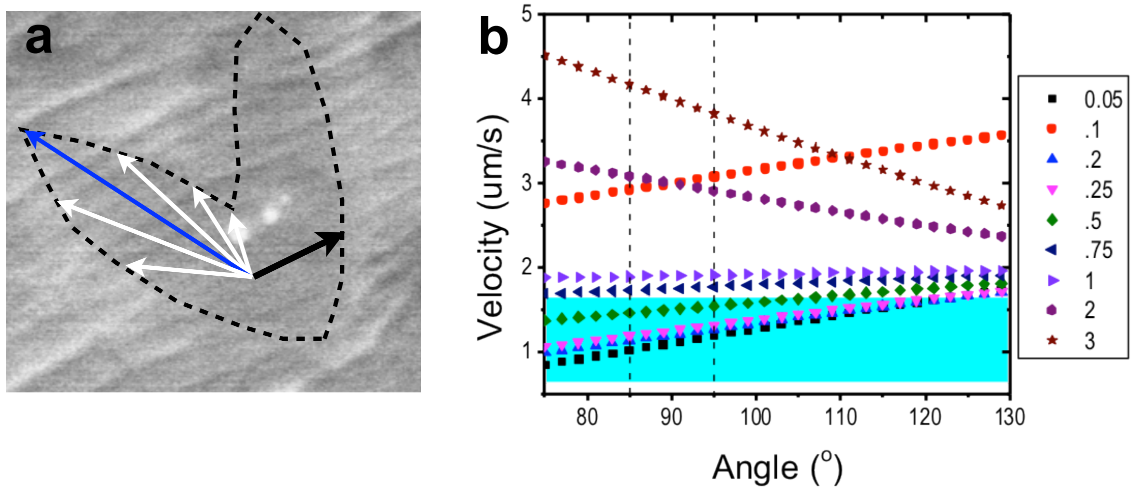

Supplementary Figure S1. Angular velocity dependence calculations. Based on equation (1) in the manuscript where θ is determined from (a) and iterations are performed on (1) to establish which value of r is the best fit for the sharp tipped lobe. From (b), the fit prescribes a value of 0.25 for r, between 85° and 95°, which corresponds to the purely fast axis velocity calculated to be 1.13±0.67 μm (s.d.), which is represented by the blue arrow in (a).

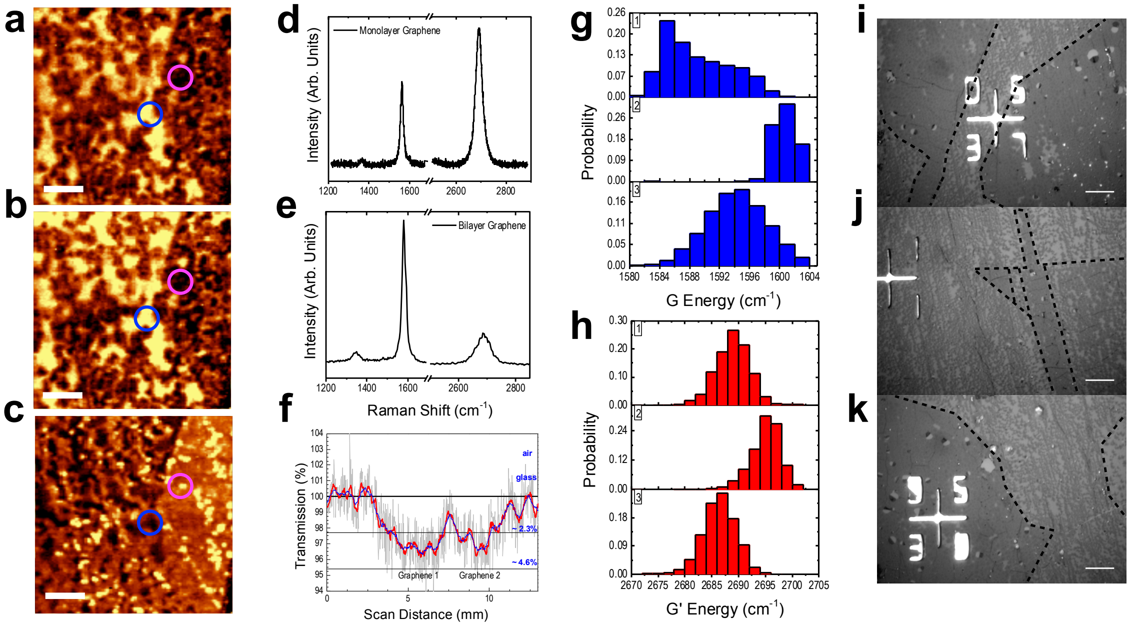

Supplementary Figure S2. (a-c) Raman maps of the G′ band FWHM, G′ and G intensities, respectively, from the area indicated by the black square in Figure 5c of the manuscript. (d) Raman spectra of monolayer graphene from the region indicated by the blue circle in (a-c). Of note is G′ FWHM = 28 cm^{-1} and $I_{G'}/I_G$ = 1.98 confirming the presence of monolayer. (e) Raman spectra of bilayer graphene from the region indicated by the purple circle in (a-c). The G′ band has broadened such that the G′ FWHM is 48 cm^{-1} and $I_{G'}/I_G < 0.5$. (f) Transmission spectra of 532 nm laser through a bilayer/monolayer sample from the same growth run in Figure 5c. The signal falls between 2.3% and 4.6% absorption proving presence of bilayer in the sample. (g, h) G and G′ band energy histograms extracted from Raman maps from the sample in Fig. 5c of the manuscript. (i-k) Additional optical images of transferred 1 min growth graphene detailing Cu grain boundaries (dashed lines) by examination of nucleation density and bilayer domain coverage.

Supplementary Table S1 EBSD details for Cu indices in Fig. 1 of the manuscript

**Figure 1** Cu Grain	Surface Normal Orientation	Unit Cell	Inverse Pole Figure	Kikuchi Lines
1	(010)	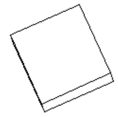	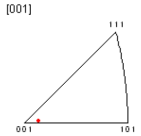	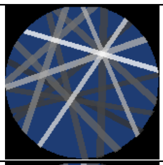
2	(00-1)	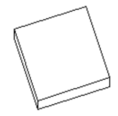	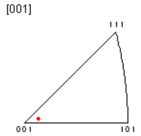	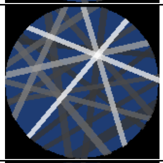
3	(010)	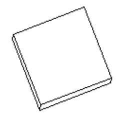	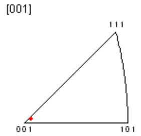	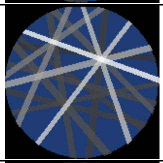
4	(00-1)	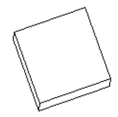	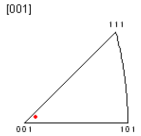	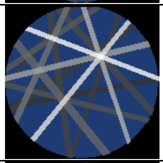
5	(00-1)	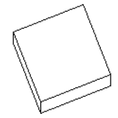	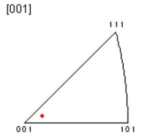	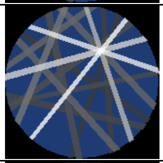
6	(00-1)	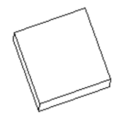	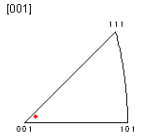	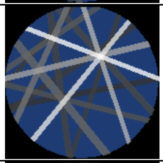
7	(00-1)	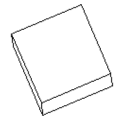	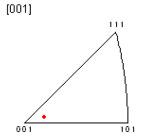	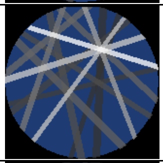
8	(010)	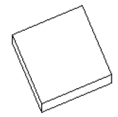	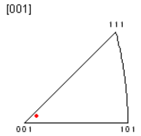	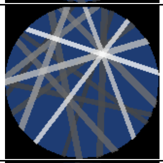
9	(010)	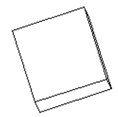	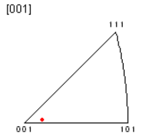	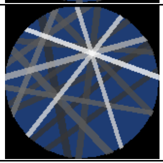

Supplementary Table S2 EBSD details for Cu indices in Fig. 3a of the manuscript

**Figure 3a** Cu Grain	Surface Normal Orientation	Unit Cell	Inverse Pole Figure	Kikuchi Lines
1	(-100)	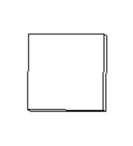	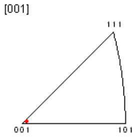	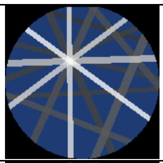
2	(010)	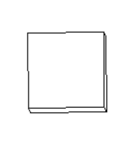	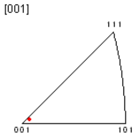	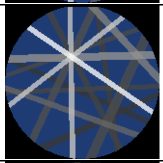
3	(010)	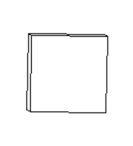	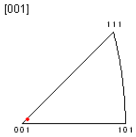	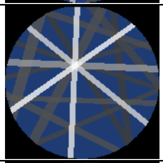
4	(-13-7)	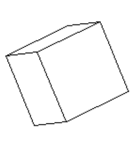	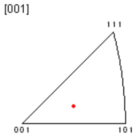	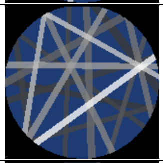
5	(173)	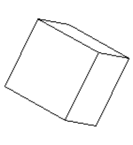	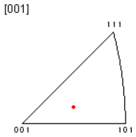	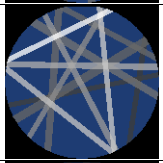
6	(-13-7)	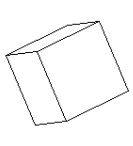	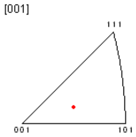	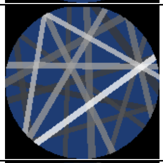
7	(173)	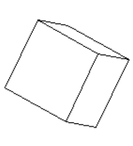	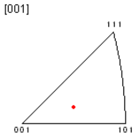	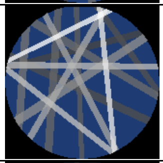
8	(00-1)	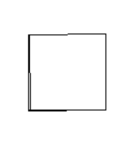	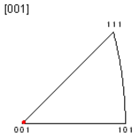	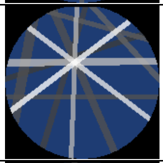
9	(001)	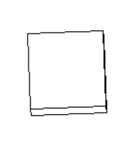	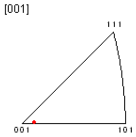	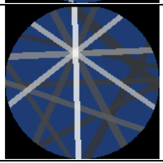

Supplementary Table S3 EBSD details for Cu indices in Fig. 3d of the manuscript

**Figure 3d**<br>Cu Grain	Surface Normal Orientation	Unit Cell	Inverse Pole Figure	Kikuchi Lines
1	(110)	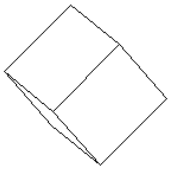	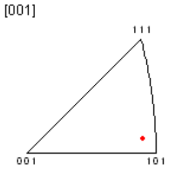	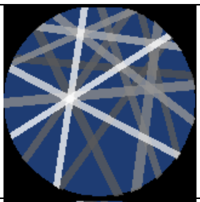
2	(112)	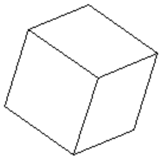	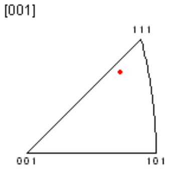	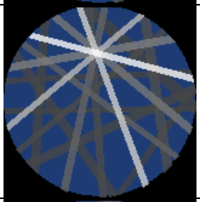
3	(122)	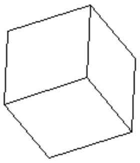	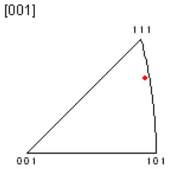	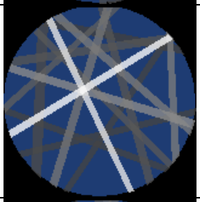
4	(122)	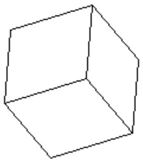	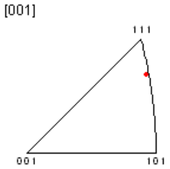	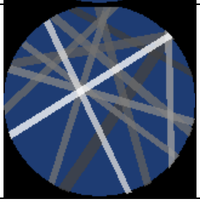

Supplementary Table S4. Means and standard deviations of orientation data in Fig. 1 and 2 of the manuscript

Data	Mean (°)	Standard Dev (°)	Probability
High symmetry angle variation from [00-1] on (010)	0.04	2.17	0.56
High symmetry angle variation from [-101] on (010)	136.97	6.30	0.44
High symmetry angle variation from [0-10] on (00-1)	2.40	7.82	0.72
High symmetry angle variation from [-110] on (00-1)	135.82	4.57	0.28
Angle between fast growth axes – 12 s	40.90	9.19	1.00
Angle between fast growth axes – 25 s	86.60	16.29	1.00

Supplementary Table S5. Means and standard deviations of Raman data in Fig. 4 of the manuscript

Cu Orientation	G Energy (cm^{-1})		G' Energy (cm^{-1})		G' FWHM (cm^{-1})		$I_{G'}/I_G$	
	Mean	Std Dev	Mean	Std Dev	Mean	Std Dev	Mean	Std Dev
(122)	1595.3	10.1	2674.6	8.8	27.8	4.3	2.05	0.44
(100)	1583.5	6.5	2689.6	10.9	28.8	5.8	1.92	0.46

Supplementary Table S6. Means and standard deviations of Raman data in Fig. 5 of the manuscript

Region	G Energy (cm^{-1})		G' Energy (cm^{-1})		G' FWHM (cm^{-1})		$I_{G'}/I_G$	
	Mean	Std Dev	Mean	Std Dev	Mean	Std Dev	Mean	Std Dev
1	1589.1	12.4	2688.8	8.2	41.8	8.3	1.59	0.53
2	1586.7	9.8	2691.0	9.9	44.2	7.8	1.60	0.47
3	1583.6	8.1	2692.2	12.6	53.5	6.9	1.28	0.52
